# Utility of carotid duplex ultrasonography in a general inner-city hospital

**DOI:** 10.1186/1476-7120-12-48

**Published:** 2014-11-25

**Authors:** Mihir Barvalia, David Silber, Michael DiVita, Abinash Joshi, Najam Wasty, Marc Cohen

**Affiliations:** Department of Medicine, Newark Beth Israel Medical Center, 201 Lyons Avenue at Osborne Terrace, Newark, NJ USA; Division of Cardiology, Newark Beth Israel Medical Center, Newark, NJ USA

**Keywords:** Carotid ultrasound, Cerebrovascular disease, Utilization

## Abstract

**Background:**

Carotid Duplex Ultrasonography (CDUS) is one of the non-invasive imaging modalities used to evaluate for carotid artery stenosis. However, it is often used in patients with coronary artery disease (CAD), peripheral artery disease (PAD), before heart surgery, syncope and non-specific neurological symptoms although its value is unclear. Our study aimed to further investigate the yield of CDUS in these conditions.

**Methods:**

A retrospective analysis was conducted on 827 consecutive carotid ultrasounds ordered between March 2013 and August 2013 at Newark Beth Israel Medical Center. Clinical characteristics such as age, sex, smoking status, systemic hypertension, diabetes mellitus, CAD, PAD, carotid bruit and indications for carotid ultrasound were included. Significant cerebrovascular disease (sCBVD) was defined as greater than or equal to 50% diameter reduction in internal carotid arteries (ICA) or any degree of occlusion in vertebrobasilar system.

**Results:**

Only 88 out of 827 (10.6%) patients had sCBVD. Using logistic regression analysis we identified age greater than 65 years (OR 2.1, 95% CI 1.2 to 3.7; P = 0.006), carotid bruit (OR 7.8, 95% CI 3.6 to 16.6; P <0.001) and history of prior carotid endarterectomy or carotid artery stenting (OR 5.8, 95% CI 2.3 to 14.8; P <0.001) as significant predictors of sCBVD.

**Conclusions:**

Significant carotid artery stenosis is more likely in patients 65 years and older, presence of carotid bruit and prior CEA. On the other hand, it has low diagnostic yield in less than 65-year-old individuals, syncope and non-focal neurological symptoms. This highlights the need for better risk prediction models in order to promote optimal utilization.

## Introduction

Carotid artery stenosis is an important cause of cervical bruit, amaurosis fugax, acute ischemic stroke and transient ischemic attack (TIA). These patients can be evaluated with carotid duplex ultrasonography. Prevalence of carotid artery stenosis in the general population is 0.2% to 7.5% in moderate (>50%) stenosis and lower 0% to 3.1% in severe (>70%) stenosis [1]. However, only selected asymptomatic individuals with severe carotid artery stenosis greater than 70% are shown to benefit from carotid endarterectomy (CEA) [2]. While CEA reduces incidence of stroke [2], routine use of CDUS has not been shown to reduce peri-operative stroke or mortality [3]. CDUS is often used in patients with planned Coronary Artery Bypass Grafting (CABG), known atherosclerosis (coronary or peripheral artery disease), and syncope. Although studies [4, 5] have shown correlation of carotid artery disease and coronary artery disease, its utility in asymptomatic patients is unclear [6]. In 2011 American College of Cardiology Foundation (ACCF) delineated recommendations for appropriate use of CDUS [7]. Our objective is to determine the yield of CDUS and identify risk factors of significant cerebrovascular disease (sCBVD). We also seek to calculate prevalence of significant carotid artery disease among appropriate and uncertain indications as per ACCF appropriate use criteria task force. Our hypothesis is that CDUS is over utilized for uncertain or inappropriate indications at our institution. We hope that our study helps providers in recognizing this and thereby promote optimal utilization of carotid ultrasound.

## Materials and methods

### Study design and patients

After approval of Institutional Review Board at Newark Beth Israel Medical Center, we included 827 carotid ultrasounds consecutively ordered between March 1st, 2013 and August 31st, 2013 at Newark Beth Israel Medical Center. Baseline characteristics such as age, sex, diabetes mellitus, systemic hypertension, coronary artery disease (CAD), peripheral arterial disease (PAD), smoking history, carotid bruit and indications for CDUS were abstracted using retrospective chart review. Importantly, our data also included a cohort of patients who underwent CDUS before CABG (91), valvular replacement/repair (34) and Orthotopic Heart Transplantation (25).

### Study protocol

All studies were performed in vascular laboratory using duplex scanner GE Logiq E-9 by credentialed vascular technologists. Patients were asked to lay supine with chin raised and head slightly turned. Imaging started with common carotid artery (CCA) in transverse position at clavicular level followed by examination in a sagittal plane utilizing gray scale. Then, spectral waveform analysis was obtained with transducer in sagittal plane and doppler angle kept between 45 and 60 degrees positioned in proximal CCA. This procedure was repeated along entire length of CCA and a representative spectral waveform was recorded of the proximal, mid and distal CCA and bulb area. Doppler samples and representative spectral waveforms were recorded of proximal, mid and distal internal carotid artery (ICA) and proximal external carotid artery (ECA). If any plaque was noted, waveforms were recorded in area proximal to plaque, at the area of plaque and distal to the plaque. Similarly, if there was significant stenosis waveform were recorded proximal to stenosis, at the stenosis and distal to stenosis if they were not already documented as per stated protocol. Finally, waveforms of vertebral and subclavian arteries were recorded. Peak systolic velocities, end diastolic velocities, plaque description using B-mode was also documented.

Stenosis in vertebrobasilar system or greater than 50% ICA stenosis was considered significant cerebrovascular disease (sCBVD) as per the Intersocietal Accreditation Commission (IAC) vascular testing carotid stenosis interpretation criteria based on Society of Radiologists in Ultrasound Consensus Conference [8]. Based on these guidelines less than 50% ICA stenosis was diagnosed with peak systolic velocity (PSV) less than 125 cm/sec and no visible plaque or intimal thickening, 50%–69% stenosis was identified when ICA PSV was 125–230 cm/sec with visible plaque and ICA PSV greater than 230 cm/sec along with visible plaque and lumen narrowing was comparable with 70% stenosis to near occlusion. Markedly narrowed lumen was suggestive of near occlusion and no detectable lumen or flow at gray-scale was indicative of total occlusion. These generalized thresholds were used to minimize variability in reporting of degree of stenosis. All results were read and confirmed by 2 board certified vascular surgeons to reduce inter-observer variability.

### Statistical analysis

Statistical analysis was completed using SPSS software, version 22.0. Continuous data was shown using median and interquartile range (IQR) while categorical data was shown in frequencies and percentages. Age, sex, smoking status, CAD, PAD, systemic hypertension, diabetes mellitus, TIA, acute ischemic stroke, carotid bruit, cardiac surgery, syncope, and prior carotid endarterectomy (CEA) or carotid artery stenting (CAS) were fitted as independent variables and sCBVD was fitted as a dependent variable. A multinomial step-wise logistic regression was then performed along with likelihood ratio, odds ratio and 95% confidence intervals to determine association of various risk factors with sCBVD. A P-value of less than 0.05 was considered statistically significant.

## Results

Out of 827 CDUS performed, significant cerebrovascular disease (sCBVD) was found in 88 (10.6%) cases. Baseline characteristics and demographics are shown in Table 1. 62 (7.5%) patients were found to have 50-69% ICA occlusion, 11 (1.3%) had 70-99% ICA occlusion, 3 (0.4%) patients had near total occlusion, 10 (1.2%) patients had total ICA occlusion and 2 (0.2%) had vertebral artery occlusion. 7 individuals with severe ICA stenosis (70-99%) underwent carotid endarterectomy while 4 were treated with medical therapy. Thus, 11 out of 827 (1.3%) patients had 70-99% stenosis in which a carotid intervention was deemed beneficial.

**Table 1:** **Baseline clinical characteristics and demographics**

Variables	Total	sCBVD	No sCBVD	% sCBVD
	N (%) = 827 (100)	n (%) = 88 (10.6)	n (%) = 739 (89.4)	
Age				
Median	67	65	67	
IQR	56-77	62-82	57-77	
≥ 65	481 (58.2)	66 (75.0)	415 (56.2)	13.7
< 65	346 (41.8)	22 (25.0)	324 (43.8)	6.4
Sex				
Female	442 (53.4)	50 (56.8)	392 (53.0)	11.3
Male	385 (46.6)	38 (43.2)	347 (47.0)	9.9
DM	299 (36.2)	36 (40.9)	263 (35.6)	12
HTN	400 (48.4)	48 (54.5)	352 (47.6)	12
Smoker	164 (9.8)	22 (25)	142 (19.2)	13.4

**Figure 1:**
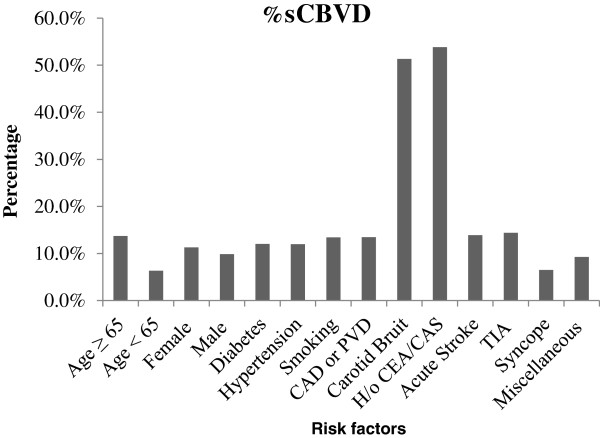
**Percentage of sCBVD across all risk factors.** Legend: sCBVD – significant cerebrovascular disease. H/o CEA/CAS – history of prior carotid endarterectomy or carotid artery stenting but asymptomatic and without carotid bruit. CAD/PAD – Coronary Artery Disease or Peripheral Artery Disease. DM – Diabetes Mellitus. HTN – systemic hypertension. TIA – Transient Ischemic Attack. Miscellaneous – non-focal neurological symptoms and before cardiac surgery. OHT – Orthotopic Heart Transplantation.

Yield of carotid ultrasound was different among various indications (Table 2 and Figure 1). Prevalence of sCBVD was higher in all appropriate use indications when compared to uncertain use indications except in patients undergoing heart transplant (Table 2). Patients with age ≥65 (OR 2.1, 95% CI 1.2-3.7; P = 0.006), carotid bruit (OR 7.7, 95% CI 3.6-16.6; P <0.001) and prior carotid endarterectomy (CEA) or prior carotid artery stenting (CAS) (OR 5.8, 95% CI 2.3-14.8; P <0.001) were significantly more likely to have sCBVD (Table 3). Out of 26 patients with previous carotid endarterectomy, sCBVD was seen in 14 patients. Among 14 cases, contralateral ICA stenosis was seen in 11 patients with 50-69% stenosis in 3 patients, 70-99% stenosis in 5 patients and absent flow in 3 patients. Remaining 3 out of 14 patients had stenosis in ipsilateral ICA. Mean peak systolic velocities (PSV) are listed in Table 4. Average PSV was 228.9 cm/s and 556.1 cm/s in patients with 50-69% stenosis and 70-99% stenosis respectively.

**Table 2:** **Prevalence of sCBVD in appropriate use and uncertain use indications**

Indications	Total	sCBVD	No sCBVD	% sCBVD
	N (%) = 827 (100)	n (%) = 88 (10.6)	n (%) = 739 (89.4)	
**Appropriate**				
Bruit	37 (4.5)	19 (21.6)	18 (2.4)	51.4
H/o CEA/CAS	26 (3.1)	14 (15.9)	12 (1.6)	53.8
Acute stroke	72 (8.7)	10 (11.4)	62 (8.4)	13.9
TIA	125 (15.1)	18 (20.5)	107 (14.5)	14.4
CAD or PAD	394 (49.6)	53 (60.2)	341 (46.1)	13.5
**Uncertain**				
Syncope	184 (22.2)	12 (13.6)	172 (23.3)	6.5
Non-focal neurological symptoms	33 (4)	2 (2.3)	31 (4.2)	6.1
Before cardiac surgery				
a. CABG	91 (11)	9 (10.2)	82 (11.1)	9.9
b. Valvular	34 (4.1)	2 (2.3)	32 (4.3)	5.9
c. OHT	25 (3)	4 (4.5)	21 (2.8)	16

Legend:

• sCBVD – significant cerebrovascular disease.

• H/o CEA/CAS – history of prior carotid endarterectomy or carotid artery stenting but asymptomatic and without carotid bruit.

• CAD/PAD – Coronary Artery Disease or Peripheral Artery Disease.

• DM – Diabetes Mellitus.

• HTN – systemic hypertension.

• TIA – Transient Ischemic Attack.

• Non focal neurological symptoms include delirium, gait abnormality or paresthesias.

• OHT – Orthotopic Heart Transplantation.

• CABG – Coronary Artery Bypass Grafting.

**Table 3:** **Risk factors predictive of sCBVD using logistic regression, odds ratio and 95% CI**

Risk factors	OR (Odds ratio)	95% CI interval		P-value
Bruit	7.75	3.57	16.83	<0.001
H/o CEA/CAS	5.77	2.27	14.65	<0.001
Age ≥65	2.14	1.24	3.68	0.006
CAD/PAD	1.36	0.81	2.3	0.25
TIA	1.54	0.72	3.27	0.26
DM	1.28	0.78	2.12	0.34
Syncope	0.77	0.39	1.52	0.45
Cardiac surgery	0.64	0.15	2.83	0.56
Sex	0.87	0.53	1.43	0.58
Smoker	1.04	0.57	1.91	0.9
Stroke	1.05	0.41	2.71	0.92
HTN	0.99	0.58	1.67	0.96

**Table 4:** **Peak systolic velocities and degree of stenosis in 88 cases with sCBVD**

Stenosis	PSV (cm/s)	N(%)
50-69%	228.9	62 (7.5%)
70-99%	556.1	11 (1.3%)
Near occlusion	Undetectable	3 (0.4%)
Total occlusion	Undetectable	10 (1.2%)
Vertebral	-	2 (0.2%)

Legend:

• sCBVD – significant cerebrovascular disease.

• H/o CEA/CAS – history of prior carotid endarterectomy or carotid artery stenting but asymptomatic and without carotid bruit.

• CAD/PAD – Coronary Artery Disease or Peripheral Artery Disease.

• TIA – Transient Ischemic Attack.

• DM – Diabetes Mellitus.

• HTN – systemic hypertension.

CDUS had lowest yield in patients younger than 65 years (6.4%), presenting with syncope (6.5%) non-focal neurologic symptoms (6.1%) such as delirium, gait imbalance, paresthesias and patients undergoing valvular repair or replacement (5.9%). Of note, sCBVD was higher (16%) in patients undergoing Orthotopic Heart Transplantation or Left Ventricular Assist Device placement but didn’t reach statistical significance.

## Discussion

Interestingly, prevalence of severe carotid artery stenosis (≥70%) was lower (1.3%) in our study considering it included patients with symptomatic carotid artery disease. This is similar to previous studies albeit in asymptomatic patients [1, 9]. Recent guidelines adopted by the ACCF [7] and USPSTF [9] indicate that performing "screening" CDUS in asymptomatic patients is not as useful compared to symptomatic patients. Evaluation of ischemic stroke, TIA, focal neurological symptoms, cervical bruit as well as asymptomatic patients with known CAD, PAD or abdominal aortic aneurysm was considered appropriate indications. However syncope, planned CABG and valvular surgery were labeled as an "uncertain" indication. Carotid intima-media thickness [6] and computerized tomography guided coronary artery calcium scoring are two of the most commonly used techniques to detect subclinical atherosclerosis [10, 11]. However, carotid duplex ultrasound exam does not include formal measurement of carotid intimal medial thickness. In addition, several studies support the association of CAD and carotid artery disease [4, 10] as well as between PAD and carotid artery disease [12, 13]. Thus, patients with CAD or PAD may warrant assessment of carotid artery disease even in the absence of any focal neurological symptoms. Carotid bruit can also signify systemic atherosclerosis [14] and thus impart increased risk of stroke [15], TIAs and mortality [16]. Some studies indicate the risk of stroke after cardiac surgery to be about 2% in patients undergoing CABG [17, 18]. Factors such as advanced age, presence of PAD, left main disease, diabetes mellitus, systemic hypertension, stroke and history of previous cerebrovascular disease could confer higher risk of peri-operative stroke and thus suggest the need for screening CDUS [17]. While a few studies have demonstrated correlation between these risk factors and carotid artery stenosis ≥50% [19, 20] these risk prediction models are not well validated. Moreover, there are no randomized controlled trials to support performing CEA in asymptomatic individuals prior to vascular surgeries such as CABG and valvular repair/replacement. Routine use of CDUS before heart transplantation is also unclear.

Several factors can lead to overutilization of carotid ultrasound such as ease of availability, lack of conclusive evidence supporting CDUS in asymptomatic individuals and concerns for peri-operative stroke. To date there is no conclusive evidence that routinely screening asymptomatic individuals for carotid artery stenosis reduces peri-operative stroke. In symptomatic patients, CDUS can detect significant carotid artery stenosis in order to reduce risk of peri-operative stroke via medical therapy or surgical intervention. Three RCTs comparing carotid endarterectomy with medical therapy showed 27% reduction (RR 0.72, 95% CI 0.58-0.90) of stroke 30 days post CEA [21]. Recent advances in medical therapy with higher statin use has also reduced incidence of ipsilateral stroke to about 1.13% per year [21, 22]. ACST-1 trial included patients without stroke, TIA or any other relevant neurological symptoms in past 6 months [2]. However, we found that non-focal neurological symptoms were a common indication to perform CDUS. There is no strong evidence supporting such rationale.

There are several limitations in our study. There could be inter-observer variability among technologists. Severity of CAD could affect degree of atherosclerosis in carotid vasculature and thereby could be a significant predictor of cerebrovascular disease. It is likely that bruit on physical exam was under-documented or examined only in patients with high risk for atherosclerotic cardiovascular disease. This could explain high yield of carotid stenosis in such patients. Moreover, patient with less than 50% stenosis of carotid arteries could have heterogeneous unstable plaque and thus could be considered high risk for stroke. High PSV in patients with previous CEA could be attributed to CEA however most patients with sCBVD in this group had contralateral ICA stenosis. Being a single center study results need to be confirmed in a multi-center prospective randomized study in order to apply it in a much broader population. Although our study shows few risk predictors of sCBVD, its accuracy needs to be studied in a validation cohort. On the other hand, our data is different from previous studies [17, 20] as it includes a cohort of patients awaiting heart transplantation.

## Conclusions

Yield of carotid ultrasound in patients less than 65 years age, syncope, non-focal neurological symptoms and prior to cardiac surgeries was low. Reducing its use in low risk patients can eliminate unnecessary use of CDUS and thus optimize efficient utilization. On the contrary, certain patients may have higher risk of stroke despite non-significant carotid artery stenosis such as those with unstable carotid plaque. Hence, a case-by-case approach may be prudent and CDUS may be needed in such scenarios. Finally, randomized prospective study and a cost-effectiveness analysis can provide better risk prediction model for detecting significant carotid artery stenosis in both high and low risk patients.

### Consent

Since this was a retrospective chart review, all health information was de-identified in compliance with IRB and U.S. Department of Health and Human Services Privacy Rule. We obtained a waiver of HIPAA (Health Insurance Portability and Accountability Act) privacy authorization, as it was impractical to conduct research without the waiver.
